# Supplementary material for: Anti-PD-L1-Based Bispecific Antibodies Targeting Co-Inhibitory and Co-Stimulatory Molecules for Cancer Immunotherapy
Source: Molecules. 2024 Jan 17;29(2):454. doi: 10.3390/molecules29020454 (PMC10819708; doi:10.3390/molecules29020454)
Supplement: Supplementary file 1 [file molecules-29-00454-s001.zip › molecules-2737952-supplementary.pdf]

Supporting information

Anti-PD-L1 Based Bispecific Antibodies Targeting Co-Inhibitory and Co-Stimulatory Molecules For Cancer Immunotherapy

Qiaohong Geng[a,b]* and Peifu Jiao[a]*

[a] School of Chemistry and Chemical Engineering, Qilu Normal University, Jinan, Shandong 250200, China

[b] Shandong Shining Pharm Co.,Ltd., Jinan, Shandong 250102, China

* Correspondences: Gengqh2006@163.com (Q.G.); Jiaopf2006@163.com (P.J.)

Table S1 Detailed sequence information of each anti-PD-L1 based bsAbs.

BsAb	Targets	Sequence
BCP-84	CTLA-4×PD-L1	Heavy chain fusion polypeptide<br>EVQLVESGGGLVQPGGSLRLSCAASGYTYSRHCLGWFRQAPGKGREAVSTIDSDGSTSYADSVKGRF<br>TISRDNAKNTLYLQMNSLRPEDTAVYYCAIGPNPRYCSGAPNTRGAEHYFGYWGQGTLVTVSSEPKS<br>SDKTHTSPPSPEVQLVESGGGLVQPGGSLRLSCAASGFTFSDSWIHWVRQAPGKGLEWVAWISPYGG<br>STYYADSVKGRFTISADTSKNTAYLQMNSLRAEDTAVYYCARRHWPGGFDYWGQGTLVTVSSAST<br>KGPSVFPLAPSSKSTSGGTAALGCLVKDYFPEPVTVSWNSGALTSGVHTFPAVLQSSGLYSLSSVVTV<br>PSSSLGTQTYICNVNHKPSNTKVDKKVEPKSCDKTHTCPPCPAPELLGGPSVFLFPPKPKDTLMISRTP<br>EVTCVVVDVSHEDPEVKFNWYVDGVEVHNAKTKPREEQYASTYRVVSVLTVLHQDWLNGKEYKC<br>KVSNKALPAPIEKTISKAKGQPREPQVYTLPPSREEMTKNQVSLTCLVKGFYPSDIAVEWESNGQPEN<br>NYKTTPPVLDSDGSFFLYSKLTVDKSRWQQGNVFSCSVMHEALHNHYTQKSLSLSPG<br>Light chain<br>DIQMTQSPSSLSASVGDRVTITCRASQDVSTAVAWYQQKPGKAPKLLIYSASFLYSGVPSRFSGSGSG<br>TDFTLTISSLQPEDFATYYCQQYLYHPATFGQGTKVEIKRTVAAPSVFIFPPSDEQLKSGTASVVCLLN<br>NFYPREAKVQWKVDNALQSGNSQESVTEQDSKDSTYSLSSTLTLSKADYEKHKVYACEVTHQGLSS<br>PVTKSFNRGEC
BCP-85	CTLA-4×PD-L1	Heavy chain fusion polypeptide<br>EVQLVESGGGLVQPGGSLRLSCAASGDSPSVNYMGWFRQAPGKGREEVSSIYPTGGTFYTDSVKGRF<br>TISRDNAKNTLYLQMNSLRPEDTAVYYCAAGKWGTDYWGQGTLVTVSSEPKSSDKTHTSPPSPEVQ<br>LVESGGGLVQPGGSLRLSCAASGFTFSDSWIHWVRQAPGKGLEWVAWISPYGGSTYYADSVKGRFTI<br>SADTSKNTAYLQMNSLRAEDTAVYYCARRHWPGGFDYWGQGTLVTVSSASTKGPSVFPLAPSSKST<br>SGGTAALGCLVKDYFPEPVTVSWNSGALTSGVHTFPAVLQSSGLYSLSSVVTVPSSSLGTQTYICNVN<br>HKPSNTKVDKKVEPKSCDKTHTCPPCPAPELLGGPSVFLFPPKPKDTLMISRTPEVTCVVVDVSHEDP<br>EVKFNWYVDGVEVHNAKTKPREEQYASTYRVVSVLTVLHQDWLNGKEYKCKVSNKALPAPIEKTIS<br>KAKGQPREPQVYTLPPSREEMTKNQVSLTCLVKGFYPSDIAVEWESNGQPENNYKTTPPVLDSDGSF<br>FLYSKLTVDKSRWQQGNVFSCSVMHEALHNHYTQKSLSLSP<br>Light chain<br>DIQMTQSPSSLSASVGDRVTITCRASQDVSTAVAWYQQKPGKAPKLLIYSASFLYSGVPSRFSGSGSG<br>TDFTLTISSLQPEDFATYYCQQYLYHPATFGQGTKVEIKRTVAAPSVFIFPPSDEQLKSGTASVVCLLN<br>NFYPREAKVQWKVDNALQSGNSQESVTEQDSKDSTYSLSSTLTLSKADYEKHKVYACEVTHQGLSS<br>PVTKSFNRGEC
AB-04	CTLA-4×PD-L1	Anti-PD-L1 Variable heavy chain<br>QVQLQESGPGLVKPSETLSITCTVSGFSLSNYDISWIRQPPGKGLEWLGVIWTGGATNYNPALKSRLTI<br>SRDNSKNQVSLKMSSVTAADTAVYYCVRDSNYRYDEPFTYWGQGTLVTVSS

		Anti-PD-L1 Variable light chain<br>EIVLTQSPDTLSVTPKEKVTLTCRASQSIGTNIHWFQQKPGQSPKLLIKYASESISGVPSRFSGSGSGTD<br>FTLTINSVEAEDAATYYCQQSNSWPYTFGQCTKLEIK<br>Anti-CTLA-4 Variable heavy chain<br>QVQLVESGGGVVQPGRSLRLSCAASGFTFSSYTMHWVRQAPGKCLEWVTFISYDGNNKYYADSVK<br>GRFTISRDNSKNTLYLQMNSLRAEDTAIYYCARTGWLGPFDYWGQGTLVTVSS<br>Anti-CTLA-4 Variable light chain<br>EIVLTQSPGTLSLSPGERATLSCRASQSVGSSYLAWYQQKPGQAPRLLIYGAFSRATGIPDRFSGSGSG<br>TDFTLTISRLEPEDFAVYYCQQYGSSPWTFGCGTKVEIK
KN-046	CTLA-4×PD-L1	QVQLVESGGGLVQPGGSLRLSCAASGKMSSRRCMAWFRQAPGKERERVAKLLTTSGSTYLADSVK<br>GRFTISRDNSKNTVYLQMNSLRAEDTAVYYCAADSFEDPTCTLVTSSGAFQYWGQGTLVTVSSGAP<br>QVQLVESGGGLVQPGGSLRLSCAASGYIYSAYCMGWFRQAPGKGLEGVAAIYIGGGSTYYADSVKG<br>RFTISRDNSKNTLYLQMNSLRAEDTAVYYCAADVIPTETCLGGSWSGPFGYWGQGTLVTVSSGSEPK<br>SSDKTHTCPPCPAPELLGGPSVFLFPPKPKDTLMISRTPEVTCVVVDVSHEDPEVKFNWYVDGVEVH<br>NAKTKPREEQYNSTYRVVSVLTVLHQDWLNGKEYKCKVSNKALPAPIEKTISKAKGQPREPQVYTLP<br>PSRDELTKNQVSLTCLVKGFYPSDIAVEWESNGQPENNYKTTPPVLDSDGSFFLYSKLTVDKSRWQQ<br>GNVFSCSVMHEALHNHYTQKSLSLSPGK
PR-001573	CTLA-4×PD-L1	Polypeptide chain 1<br>DIQMTQSPSTLSASVGDRVTVTCRASQSIYIWLAWYQQKPGKAPNLLIYKASSLETGVPSRFSGSGSG<br>TEFTLTISSLQPDDFATYYCQQYYGSSRTFGQGTKVEIKRTVAAPSVFIFPPSDEQLKSGTASVVCLLN<br>NFYPREAKVQWKVDNALQSGNSQESVTEQDSKDSTYSLSSTLTLSKADYEKHKVYACEVTHQGLSS<br>PVTKSFNRGEC<br>Polypeptide chain 2<br>EVQLVESGGGLIQPGGSLRLSCAVSGFTVSKNYMSWVRQAPGKGLEWVSVVYSGGSKTYADSVKG<br>RFTISRDNSKNTLYLQMNSLRAEDTAVYYCARAVPHSPSSFDIWGQGTMVTVSSKLEPKSSDKTHTP<br>PPPPRTEVQLVESGGGLVQPGGSLRLSCAASGFTFSSYWMSWVRQAPGKGLEWVANIKQEGSEKYY<br>VDSVKGRFTISRDNAKNSLYLQMNSLRAEDTAVYYCARDRAVAGAFDIWGQGTMVTVSSASTKGPS<br>VFPLAPSSKSTSGGTAALGCLVKDYFPEPVTVSWNSGALTSGVHTFPAVLQSSGLYSLSSVVTVPSSS<br>LGTQTYICNVNHKPSNTKVDKKVEPKSCDKTHTCPPCPAPELLGGPSVFLFPPKPKDTLMISRTPEVTC<br>VVVDVSHEDPEVKFNWYVDGVEVHNAKTKPREEQYNSTYRVVSVLTVLHQDWLNGKEYKCKVSN<br>KALPAPIEKTISKAKGQPREPQVYTLPPSREEMTKNQVSLTCLVKGFYPSDIAVEWESNGQPENNYKT<br>TPPVLDSDGSFFLYSKLTVDKSRWQQGNVFSCSVMHEALHNHYTQKSLSLSPGK
FS118	LAG-3×PD-L1	Heavy chain<br>EVQLVESGGGLVQPGRSLRLSCAASGFTFDDYAMHWVRQTPGKGLEWVSGISWKSNIIGYADSVKG<br>RFTISRDNAKNSLYLQMNSLRAEDTALYYCARDITGSGSYGWFDPWGQGTLVTVSSASTKGPSVFPL<br>APSSKSTSGGTAALGCLVKDYFPEPVTVSWNSGALTSGVHTFPAVLQSSGLYSLSSWTVPSSSLGT<br>QTYICNVNHKPSNTKVDKKVEPKSCDKTHTCPPCPAPEAAGGPSVFLFPPKPKDTLMISRTPEVTCVV<br>VDVSHEDPEVKFNWYVDGVEVHNAKTKPREEQYNSTYRVVSVLTVLHQDWLNGKEYKCKVSNKA<br>LPAPIEKTISKAKGQPREPQVYTLPPSWDEPWGEDVSLTCLVKGFYPSDIAVEWESNGQPENNYKTTP<br>PVLDSDGSFFLYSKLTVPYDRVWWPDEFSCSVMHEALHNHYTQKSLSLSPG<br>Light chain<br>DIQMTQSPSSLSASVGDRVTITCRASQSISSYLNWYQQKPGKAPKPLIYVASSLQSGVPSSFSGSGSGT<br>DFTLTISSLQPEDFATYYCQQSYSNPITFGQGTRLEIKRTVAAPSVFIFPPSDEQLKSGTASVVCLLNNF<br>YPREAKVQWKVDNALQSGNSQESVTEQDSKDSTYSLSSTLTLSKADYEKHKVYACEVTHQGLSSPV

		TKSFNRGEC<br>CDR1 variable heavy chain<br>GFTFDDYA<br>CDR2 variable heavy chain<br>ISWKSNII<br>CDR3 variable heavy chain<br>ARDITGSGSYGWFDP<br>CDR1 variable light chain<br>QSISSY<br>CDR2 variable light chain<br>VAS<br>CDR3 variable light chain<br>QQSYSNPIT
ABL-501	LAG-3×PD-L1	NA
IBI323	LAG-3×PD-L1	NA
mPDL1HCv1-<br>E-sLAG3	LAG-3×PD-L1	E-linker<br>EPKSSDKTHTSPPSP<br>Heavy chain-linker-sLAG3<br>EVQLVQSGAEVKKPGASVKVSCKASGYIFTGYGITWVRQAPGQGLEWMGEIFPRRVQTYYSEKFKG<br>RVTMTTDTSTSTAYMELRSLRSDDTAVYYCARDYDPYFALDYWGQGTTVTVSSASTKGPSVFPLAP<br>SSKSTSGGTAALGCLVKDYFPEPVTVSWNSGALTSGVHTFPAVLQSSGLYSLSSVVTVPSSSLGTQTY<br>ICNVNHKPSNTKVDKKVEPKSCDKTHTCPPCPAPELLGGPSVFLFPPKPKDTLMISRTPEVTCVVVDV<br>SHEDPEVKFNWYVDGVEVHNAKTKPREEQYASTYRVVSVLTVLHQDWLNGKEYKCKVSNKALPAP<br>IEKTISKAKGQPREPQVYTLPPSREEMTKNQVSLTCLVKGFYPSDIAVEWESNGQPENNYKTTPPVLD<br>SDGSFFLYSKLTVDKSRWQQGVFSCSVMHEALHNHYTQKSLSLSPGKEPKSSDKTHTSPPSPEVQLV<br>ESGGGLVQPGGSLRLSCAASGYTVSSYCMGWFRQAPGKGREGVSAIDSDGSVSYADSVKGRFTISKD<br>NSKNTLYLQMNSLRAEDTAVYFCAADLCWVDQDQGEYNTWGQGTLVTVSS<br>Light chain<br>DIQMTQSPSSLSASVGDRVTITCRASQDVSTAVDWYQQKPGKAPKLLIYSASYRYTGVPDRFSGSGS<br>GTDFTFTISSLQPEDIATYYCQQHYSIPFTFGQGTKLEIKRTVAAPSVFIFPPSDEQLKSGTASVVCLLN<br>NFYPREAKVQWKVDNALQSGNSQESVTEQDSKDSTYSLSSTLTLSKADYEKHKVYACEVTHQGLSS<br>PVTKSFNRGEC<br>CDR1 variable heavy chain<br>GYIFTGYGIT<br>CDR2 variable heavy chain<br>EIFPRRVQTYYSEKFKG<br>CDR3 variable heavy chain<br>DYDPYFALDY<br>CDR1 variable light chain<br>RASQDVSTAVD<br>CDR2 variable light chain<br>SASYRYT<br>CDR3 variable light chain<br>QQHYSIPFT

		Heavy chain<br>EVQLVQSGAEVKKPGASVKVSCKASGYIFTGYGITWVRQAPGQGLEWMGEIFPRRVQTYYSEKFKG<br>RVTMTTDTSTSTAYMELRSLRSDDTAVYYCARDYDPYFALDYWGQGTTVTVSSASTKGPSVFPLAP<br>SSKSTSGGTAALGCLVKDYFPEPVTVSWNSGALTSGVHTFPAVLQSSGLYSLSSVVTVPSSSLGTQTY<br>ICNVNHKPSNTKVDKKVEPKSCDKTHTCPPCPAPELLGGPSVFLFPPKPKDTLMISRTPEVTCVVVDV<br>SHEDPEVKFNWYVDGVEVHNAKTKPREEQYASTYRVVSVLTVLHQDWLNGKEYKCKVSNKALPAP<br>IEKTISKAKGQPREPQVYTLPPSREEMTKNQVSLTCLVKGFYPSDIAVEWESNGQPENNYKTTPPVLD<br>SDGSFFLYSKLTVDKSRWQQGVFSCSVMHEALHNHYTQKSLSLSPGK<br>LAG3 single domain antibody (sdAb)<br>EVQLVESGGGLVQPGGSLRLSCAASGYTVSSYCMGWFRQAPGKGREGVSAIDSDGSVSYADSVKGR<br>FTISKDNSKNTLYLQMNSLRAEDTAVYFCAADLCWVDQDQGEYNTWGQGTLVTVSS<br>sLAG3 CDR1 variable heavy chain<br>GYTVSSYCMG<br>sLAG3 CDR2 variable heavy chain<br>AIDSDGSVSYADSVKG<br>sLAG3 CDR3 variable heavy chain<br>DLCWVDQDQGEYNT
W-3669	LAG-3×PD-L1	Anti-PDL1 VHH<br>EVQLVESGGGLVQPGGSLRLSCAASGHFSNLAVNWFRQAPGKERELVAGILWSGGSTFYADSVKGR<br>FTISRGNAENMLYLQMNSLRAEDTAVYYCNTGTNWGQGTLVTVSS<br>CDR1 Anti-PDL1 VHH<br>GHFSNLAVN<br>CDR2 Anti-PDL1 VHH<br>GILWSGGSTFYADSVKG<br>CDR3 Anti-PDL1 VHH<br>GTN<br>Anti-LAG3 VHH<br>QVQLVESGGGVVQPGGSLRLSCAASGLTLSQYTMGWFRQAPGKERELVAAIHWTSSVTDYADSVY<br>GRFTISRDDSKNTGYLQMNSLRAEDTAVYYCAATHYYTHRGPFDYWGQGTLVTVSS<br>CDR1 Anti-LAG3 VHH<br>GLTLSQYTMG<br>CDR2 Anti-LAG3 VHH<br>AIHWTSSVTDYADSVY<br>CDR3 Anti-LAG3 VHH<br>THYYTHRGPF
hz7F10-hzB6	LAG-3×PD-L1	Heavy chain<br>EVQLVQSGAEVKKPGASVKVSCKASGVNIKDDYMHWVRQAPGQGLEWIGRIDPEDVETKYDPKFQ<br>GRVTITADTSTSTAYMELSSLRSEDTAVYYCARSFYSNYVNYFDQWGQGTLVTVSSASTKGPSVFPL<br>APSSKSTSGGTAALGCLVKDYFPEPVTVSWNSGALTSGVHTFPAVLQSSGLYSLSSVVTVPSSSLGTQ<br>TYICNVNHKPSNTKVDKKVEPKSCDKTHTCPPCPAPEAAGGPSVFLFPPKPKDTLMISRTPEVTCVVV<br>DVSHEDPEVKFNWYVDGVEVHNAKTKPREEQYNSTYRVVSVLTVLHQDWLNGKEYKCKVSNKAL<br>PAPIEKTISKAKGQPREPQVYTLPPSRDELTKNQVSLTCLVKGFYPSDIAVEWESNGQPENNYKTTPPV<br>LDSDGSFFLYSKLTVDKSRWQQGNVFSCSVMHEALHNHYTQKSLSLSPGGGGGSPGGGSPGGGSEV<br>QLVESGGGLVQPGGSLRLSCAASEYIGDRYCAGWFRQAPGKEREGVAMIDRHGIVRYKDSVEGRFTI

		SRNHAGNTLYLQMNSLRAEDTAVYYCAADRDPTNVIPCRPEYPDMDYWGQGTLVTVSS<br>Heavy chain<br>EVQLVQSGAEVKKPGASVKVSCKASGVNIKDDYMHWVRQAPGQGLEWIGRIDPEDVETKYDPKFQ<br>GRVTITADTSTSTAYMELSSLRSEDTAVYYCARSFYSNYVNYFDQWGQGTLVTVSSASTKGPSVFPL<br>APSSKSTSGGTAALGCLVKDYFPEPVTVSWNSGALTSGVHTFPAVLQSSGLYSLSSVVTVPSSSLGTQ<br>TYICNVNHKPSNTKVDKKVEPKSCDKTHTCPPCPAPEAAGGPSVFLFPPKPKDTLMISRTPEVTCVVV<br>DVSHEDPEVKFNWYVDGVEVHNAKTKPREEQYNSTYRVVSVLTVLHQDWLNGKEYKCKVSNKAL<br>PAPIEKTISKAKGQPREPQVYTLPPSRDELTKNQVSLTCLVKGFYPSDIAVEWESNGQPENNYKTTPPV<br>LDSDGSFFLYSKLTVDKSRWQQGNVFSCSVMHEALHNHYTQKSLSLSPGGGGGSPGGGSPGGGSEV<br>QLVESGGGLVQPGGSLRLSCAASRDSDEGASCMGWFRQAPGKEREGVAIIFNAGERTDYGDSVKGR<br>FTISRDNAKNTLYLQMNSLRAEDTAVYYCATVWCGSWVARSWGQGTLVTVSS<br>Linker<br>GGGGSPGGGSPGGGS<br>Variable heavy chain (Anti-PD-L1)<br>EVQLVESGGGLVQPGGSLRLSCAASEYIGDRYCAGWFRQAPGKEREGVAMIDRHGIVRYKDSVEGR<br>FTISRNHAGNTLYLQMNSLRAEDTAVYYCAADRDPTNVIPCRPEYPDMDYWGQGTLVTVSS<br>CDR1 variable heavy chain (Anti-PD-L1)<br>EYIGDRYCAG<br>CDR2 variable heavy chain (Anti-PD-L1)<br>MIDRHGIV<br>CDR3 variable heavy chain (Anti-PD-L1)<br>DRDPTNVIPCRPEYPDMDY<br>Constant heavy chain (Anti-LAG3)<br>ASTKGPSVFPLAPSSKSTSGGTAALGCLVKDYFPEPVTVSWNSGALTSGVHTFPAVLQSSGLYSLSSV<br>VTVPSSSLGTQTYICNVNHKPSNTKVDKKVEPKSCDKTHTCPPCPAPEAAGGPSVFLFPPKPKDTLMI<br>SRTPEVTCVVVDVSHEDPEVKFNWYVDGVEVHNAKTKPREEQYNSTYRVVSVLTVLHQDWLNGKE<br>YKCKVSNKALPAPIEKTISKAKGQPREPQVYTLPPSRDELTKNQVSLTCLVKGFYPSDIAVEWESNGQ<br>PENNYKTTPPVLDSDGSFFLYSKLTVDKSRWQQGNVFSCSVMHEALHNHYTQKSLSLSPG<br>Constant light chain (Anti-LAG3)<br>RTVAAPSVFIFPPSDEQLKSGTASVVCLLNNFYPREAKVQWKVDNALQSGNSQESVTEQDSKDSTYS<br>LSSTLTLSKADYEKHKVYACEVTHQGLSSPVTKSFNRGEC<br>Variable heavy chain (Anti-LAG3)<br>EVQLVQSGAEVKKPGASVKVSCKASGVNIKDDYMHWVRQAPGQGLEWIGRIDPEDVETKYDPKFQ<br>GRVTITADTSTSTAYMELSSLRSEDTAVYYCARSFYSNYVNYFDQWGQGTLVTVSS<br>Variable light chain (Anti-LAG3)<br>DIQMTQSPSSLSASVGDRVTITCKASENVGTYVSWFQQKPGKAPKLLIYGASNRYTGVPSRFSGSGSG<br>TDFTLTISSLQPEDFATYYCGQSYSYPYTFGQGTKLEIK<br>CDR1 variable heavy chain (Anti-LAG3)<br>DDYMH<br>CDR2 variable heavy chain (Anti-LAG3)<br>RIDPEDVETKYDPKFQG<br>CDR3 variable heavy chain (Anti-LAG3)<br>SFYSNYVNYFDQWGQ<br>CDR1 variable light chain (Anti-LAG3)

		KASENVGTYVS<br>CDR2 variable light chain (Anti-LAG3)<br>GASNRYT<br>CDR3 variable light chain (Anti-LAG3)<br>GQSYSYPYT
PB-68	LAG-3×PD-L1	Variable heavy chain region (Anti-LAG-3)<br>QVQLVQSGGGVVQPGRSLRLSCAASGFTFSSYDTHWVRQAPGKGLEWVAVISYDGSNKYYADSVK<br>GRFTISRDNSKNTLYLHMNSLRAEDTAMYYCARERGWDVFDIWGQGTTVTVSS<br>Constant heavy chain region-1 (Anti-LAG-3)<br>ASTKGPSVFPLAPSSKSTSGGTAALGCLVKDYFPEPVTVSWNSGALTSGVHTFPAVLQSSGLYSLSSV<br>VTVPSSSLGTQTYICNVNHKPSNTKVDKRV<br>Constant heavy chain region-2 (Anti-LAG-3)<br>APELGRGPSVFLFPPKPKDTLMISRTPEVTCVVVDVSHEDPEVKFNWYVDGVEVHNAKTKPREEQYN<br>STYRVVSVLTVLHQDWLNGKEYKCKVSNKALPAPIEKTISKAK<br>Constant heavy chain region-3 (Anti-LAG-3)<br>GQPREPQVYTKPPSREEMTKNQVSLKCLVKGFYPSDIAVEWESNGQPENNYKTTPPVLDSDGSFFLY<br>SKLTVDKSRWQQGNVFSCSVMHEALHNHYTQKSLSLSPGK<br>Constant heavy chain region-1 (Anti-PD-L1)<br>ASTKGPSVFPLAPSSKSTSGGTAALGCLVKDYFPEPVTVSWNSGALTSGVHTFPAVLQSSGLYSLSSV<br>VTVPSSSLGTQTYICNVNHKPSNTKVDKRV<br>Constant heavy chain region-2 (Anti-PD-L1)<br>APELGRGPSVFLFPPKPKDTLMISRTPEVTCVVVDVSHEDPEVKFNWYVDGVEVHNAKTKPREEQYN<br>STYRVVSVLTVLHQDWLNGKEYKCKVSNKALPAPIEKTISKAK<br>Constant heavy chain region-3 (Anti-PD-L1)<br>GQPREPQVYTDPPSREEMTKNQVSLTCEVKGFYPSDIAVEWESNGQPENNYKTTPPVLDSDGSFFLYS<br>KLTVDKSRWQQGNVFSCSVMHEALHNHYTQKSLSLSPGK<br>Variable heavy chain region (Anti-PD-L1)<br>EVQLVQSGAEVKKPGSSVKVSCKASGGTFSTYAISWVRQAPGQGLEWMGWIIPIFDTGNYAQKFQG<br>RVTITADKSTSTAYMELTSLRSEDTAVYYCARHDYTNTVDAFDIWGQGTMVTVSS
Hanmi bsAb	PD-1×PD-L1	NA
LY-3434172	PD-1×PD-L1	NA
anti-PD-1×PD-L1 BsAb	PD-1×PD-L1	NA
609-Fab-PD-L1-IgG4	PD-1×PD-L1	NA
IMGS-001	PD-L2×PD-L1	NA
Bi-201	PD-L2×PD-L1	QVQLVESGGGLVKPGGSLRLSCAASGHTFIIYAIGWFRQAPGKGLEFVATINWSGSMTNYADSVKGR<br>FTISRDNAKNTVYLQMNSLKPDDTAVYYCAAYVGATISTAHSRYDYWGQGTLVTVSSDKTHTCPPC<br>PAPEAAGGPSVFLFPPKPKDTLMISRTPEVTCVVVDVSHEDPEVKFNWYVDGVEVHNAKTKPREEQY<br>NSTYRVVSVLTVLHQDWLNGKEYKCKVSNKALPAPIEKTISKAKGQPREPQVYTLPPSREEMTKNQV<br>SLTCLVKGFYPSDIAVEWESNGQPENNYKTTPPVLDSDGSFFLYSKLTVDKSRWQQGNVFSCSVMHE<br>ALHNHYTQKSLSLSPGGGGGSGGGGSGGGGSGGGGSGEVQLVESGGGLVQPGGSLRLSCAASGRTF<br>SSMTIGWFRQAPGKGLEFVSGIGWTSGLTVYADSVKGRFTISRDNAKNSMYLQMNSLRAEDTAVYY<br>CAADSMNRGQFDYWGQGTLVTVSS

LY-3415244	TIM-3×PD-L1	Anti-PD-L1 Heavy chain<br>QVQLVQSGAEVKKPGSSVKVSCKASGGTFSSYAISWVRQAPGQGLEWMGGIIPIFGTANYAQKFQGR<br>VTITADKSTSTAYMELSSLRSEDTAVYYCARSPDYSPYYYYGMDVWGQGTTVTVSSASTKGPSVFPL<br>APSSKSTSGGTAALGCLVKDYFPEPVTVSWNSGALTSGVHTFPAVLQSSGLYSLKSVVTVPSSSLGTQ<br>TYICNVNHKPSNTKVDKRVEPKSCDKTHTCPPCPAPEAAGGPSVFLFPPKPKDTLMISRTPEVTCVVV<br>SVSHEDPEVKFNWYVDGVEVHNAKTKPREEQYNSTYRVVSVLTVLHQDWLNGKEYKCKVSNKALP<br>APIEKTISKAKGQPREPQVYVLPPSRDELTKNQVSLLCLVKGFYPSDIAVEWESNGQPENNYLTWPPV<br>LDSDGSFFLYSKLTVDKSRWQQGNVFSCSVMHEALHNHYTQKSLSLSPGK<br>Anti-TIM3 Heavy chain<br>EVQLLESGGGLVQPGGSLRLSCAASGFTFSSYYMSWVRQAPGKGLEWVSAISGNGKSTYYADSVKG<br>RFTISRDNSKNTLYLQMNSLRAEDTAVYYCARYYNTGFDLWGQGTLVTVSSASTKGPSVFPEAPSSK<br>STSGGTAALGCLVTDYFPEPVTVSWNSGALTSGVHTFPAVLESSGLYSLWSVVTVPSSSLGTQTYICN<br>VNHKPSNTKVDKRVEPKSCDKTHTCPPCPAPEAAGGPSVFLFPPKPKDTLMISRTPEVTCVVVSVSHE<br>DPEVKFNWYVDGVEVHNAKTKPREEQYNSTYRVVSVLTVLHQDWLNGKEYKCKVSNKALPAPIEK<br>TISKAKGQPREPQVYVYPPSRDELTKNQVSLTCLVKGFYPSDIAVEWESNGQPENNYKTTPPVLDSD<br>GSFALVSKLTVDKSRWQQGNVFSCSVMHEALHNHYTQKSLSLSPGK<br>Anti-PD-L1 Light chain<br>QSVLTQPPSASGTPGQRVTISCSGSSSNIGSNTVNWYQQLPGTAPKLLIYGNSNRPSGVPDRFSGSKSG<br>TSASLAISGLQSEDEADYYCQSYDSSLSGSVFGGGIKLTVLGQPKAAPSVTLFPPSSEELQANKATLVC<br>LISDFYPGAVTVAWKADSSPVKAGVETTTPSKQSNNKYAAESELSLTPEQWKSHRSYSCQVTHEGST<br>VEKTVAPAECS<br>Anti-TIM3 Light chain<br>DIVMTQSPSSLSASVGDGVTITCQASQDIYNYLNWYQQKPGKAPKLLIYYASSIVSGVPSRFSGSGSGT<br>DFTLTISSLQPEDFATYYCQQASSFPPTFGQGTKLEIKRTVAAPSVFIFPPSDEQLKSGTARVGCLLNNF<br>YPREAKVQWKVDNALQSGNSQESVTEQDSKDSTYSLRSALTLSKADYEKHKVYACEVTHQGLSSPV<br>TKSFNRGEC<br>Anti-PD-L1 Variable heavy chain<br>QVQLVQSGAEVKKPGSSVKVSCKASGGTFSSYAISWVRQAPGQGLEWMGGIIPIFGTANYAQKFQGR<br>VTITADKSTSTAYMELSSLRSEDTAVYYCARSPDYSPYYYYGMDVWGQGTTVTVSS<br>Anti-TIM3 Variable heavy chain<br>EVQLLESGGGLVQPGGSLRLSCAASGFTFSSYYMSWVRQAPGKGLEWVSAISGNGKSTYYADSVKG<br>RFTISRDNSKNTLYLQMNSLRAEDTAVYYCARYYNTGFDLWGQGTLVTVSS<br>Anti-PD-L1 Variable light chain<br>QSVLTQPPSASGTPGQRVTISCSGSSSNIGSNTVNWYQQLPGTAPKLLIYGNSNRPSGVPDRFSGSKSG<br>TSASLAISGLQSEDEADYYCQSYDSSLSGSVFGGGIKLTVLG<br>Anti-TIM3 Variable light cahin<br>DIVMTQSPSSLSASVGDGVTITCQASQDIYNYLNWYQQKPGKAPKLLIYYASSIVSGVPSRFSGSGSGT<br>DFTLTISSLQPEDFATYYCQQASSFPPTFGQGTKLEIK
BTP-21	TIGIT×PD-L1	AS19584VH28 sdAb-Fc (IgG4)<br>EVQLVESGGGLVQPGGSLRLSCAASGYKYGVYSMGWFRQAPGKGLEGVSAICSGGRTTYSDSVKGR<br>FTISRDNSNQILYLQMNSLRAEDTAVYYCAARPLWTGDCDLSSSWYKTWGQGTLVTVSSESKYGPP<br>CPPCPAPEFLGGPSVFLFPPKPKDTLMISRTPEVTCVVVDVSQEDPEVQFNWYVDGVEVHNAKTKPR<br>EEQFNSTYRVVSVLTVLHQDWLNGKEYKCKVSNKGLPSSIEKTISKAKGQPREPQVYTLPPSQEEMT<br>KNQVSLTCLVKGFYPSDIAVEWESNGQPENNYKTTPPVLDSDGSFFLYSRLTVDKSRWQEGNVFSCS

		VMHEALHNHYTQKSLSLSLGK<br>IgG1 hinge region<br>EPKSCDKTHTCPPCP
HLX-301	TIGIT×PD-L1	NA
HB-0036	TIGIT×PD-L1	NA
TIGIT-Fc-93-VH6	TIGIT×PD-L1	Heavy chain<br>EVKLVESGGGLVQPGGSLRLSCAASGFTFSDYYMYWVRQAPGKRLEWVASITKGGGSTYYPDTLKGRFTISRDNAKNSLYLQMNRLRAEDTAVYYCARQSSYDFVMDYWGQGTTVTVSSASTKGPSVFPLAPSSKSTSGGTAALGCLVKDYFPEPVTVSWNSGALTSGVHTFPAVLQSSGLYSLSSVVTVPSSSLGTQTYICNVNHKPSNTKVDKKVEPKSCDKTHTCPPCPAPELLGGPSVFLFPPKPKDTLMISRTPEVTCVVVDVSHEDPEVKFNWYVDGVEVHNAKTKPREEQYNSTYRVVSVLTVLHQDWLNGKEYKCKVSNKALPAPIEKTISKAKGQPREPQVYTLPPSREEMTKNQVSLTCLVKGFYPSDIAVEWESNGQPENNYKTTPPVLDSDGSFFLYSKLTVDKSRWQQGNVFSCSVMHEALHNHYTQKSLSLSPGKGGGGSGGGGSGGGGSGGGGSEVQLVESGGGLVQPGGSLRLSCAASGFTFTFRHYVMGWFRQAPGKEREFVAAISWSGSGSYYADSVKGRFTISRDNSKNTVYLQMNSLRAEDTAVYYCAADMTTRMSQASREYDYWGQGTLVTVSS<br>Light chain<br>DIVMTQSPSSLSASVGDRVTITCKASQDVDTAVAWYQQKPGKAPKLLIYWASARHTGVPSRFSGSGSGTDFTFTISSLQPEDIATYYCQQYSNYPLTFGQGTKLEIKRTVAAPSVFIFPPSDEQLKSGTASVVCLLNNFYPREAKVQWKVDNALQSGNSQESVTEQDSKDSTYSLSSTLTLSKADYEKHKVYACEVTHQGLSSPVTKSFNRGEC<br>Variable heavy chain (Anti-TIGIT)<br>EVKLVESGGGLVQPGGSLRLSCAASGFTFSDYYMYWVRQAPGKRLEWVASITKGGGSTYYPDTLKGRFTISRDNAKNSLYLQMNRLRAEDTAVYYCARQSSYDFVMDYWGQGTTVTVSS<br>Variable light chain (Anti-TIGIT)<br>DIVMTQSPSSLSASVGDRVTITCKASQDVDTAVAWYQQKPGKAPKLLIYWASARHTGVPSRFSGSGSGTDFTFTISSLQPEDIATYYCQQYSNYPLTFGQGTKLEIK<br>CDR1 variable heavy chain (Anti-TIGIT)<br>DYYMY<br>CDR2 variable heavy chain (Anti-TIGIT)<br>SITKGGGSTYYPDTLKG<br>CDR3 variable heavy chain (Anti-TIGIT)<br>QSSYDFVMDY<br>CDR1 variable light chain (Anti-TIGIT)<br>KASQDVDTAVA<br>CDR2 variable light chain (Anti-TIGIT)<br>WASARHT<br>CDR3 variable light chain (Anti-TIGIT)<br>QQYSNYPLT<br>Variable heavy chain (Anti-PD-L1)<br>EVQLVESGGGLVQPGGSLRLSCAASGFTFTFRHYVMGWFRQAPGKEREFVAAISWSGSGSYYADSVKGRFTISRDNSKNTVYLQMNSLRAEDTAVYYCAADMTTRMSQASREYDYWGQGTLVTVSS<br>CDR1 variable heavy chain (Anti-PD-L1)<br>RHYVMG<br>CDR2 variable heavy chain (Anti-PD-L1)

		AISWSGSGSYYADSVKG<br>CDR3 variable heavy chain (Anti-PD-L1)<br>DMTTRMSQASREYDY
P-O-T	TIGIT×PD-L1	Heavy chain (Anti-PDL1 and Anti-TIGIT)<br>QVQLVQSGAEVKKPGASVKVSCKASGYTFTSYWMHWVRQAPGQGLEWMGRITPSSGFAMYNEKF<br>KNRVTMTRDTSTSTVYMELSSLRSEDTAVYYCARGGSSYDYFDYWGQGTTVTVSSGGGGSSGAPRF<br>LTRPKAFVVSVGKDATLSSQIVGNPFPQVSWEKDKQPVTAGVRFRLAQDGDLYRLKILDLQLSDSGQ<br>YVSRARNAIGEAFACLGLQVDAEAGGGGSEVQLVQSGAEVKKPGASVKVSCKASGYTFTNYWMH<br>WVRQAPGQGLEWMGRIDPDSTGSKYNEKFKTRVTMTRDTSTSTVYMELSSLRSEDTAVYYCAREG<br>AYGYYFDYWGQGTLVTVSSASTKGPSVFPLAPSSKSTSGGTAALGCLVKDYFPEPVTVSWNSGALTS<br>GVHTFPAVLQSSGLYSLSSVVTVPSSSLGTQTYICNVNHKPSNTKVDKKVEPKSCDKTHTCPPCPAPE<br>LLGGPSVFLFPPKPKDTLMISRTPEVTCVVVDVSHEDPEVKFNWYVDGVEVHNAKTKPREEQYNSTY<br>RVVSVLTVLHQDWLNGKEYKCKVSNKALPAPIEKTISKAKGQPREPQVYTLPPSRDELTKNQVSLTC<br>LVKGFYPSDIAVEWESNGQPENNYKTTPPVLDSDGSFFLYSKLTVDKSRWQQGNVFSCSVMHEALH<br>NHYTQKSLSLSPGK<br>Light chain 1 (Anti-PDL1 and Anti-TIGIT)<br>DIVLTQSPASLAVSPGQRATITCRASESVSIHGTHLMHWYQQKPGQPPKLLIYAASKLESGVPARFSGS<br>GSGTDFTLTINPVEAEDTANYYCQQSFEDPLTFGQGTKLEIKGGGGSGIPPKIECLPIDISIDEGKVLTV<br>ASAFTGEPTPEVTWSTGGRKIHSQEQGRFHIENTDDLTTLIIKDVQKQDGGLYTLTLRNEFGSDSATV<br>NIHIRSI<br>Light chain 2 (Anti-PDL1 and Anti-TIGIT)<br>DIQMTQSPSSLSASVGDRVTITCRASENIYSYLAWYQQKPGKSPKLLIYNARTLAEGVPSRFSGSGSGT<br>DFTLTISSLQPEDFATYYCQYHSGSPLPFGGGTKVEIKRTVAAPSVFIFPPSDEQLKSGTASVVCLLNNF<br>YPREAKVQWKVDNALQSGNSQESVTEQDSKDSTYSLSSTLTLSKADYEKHKVYACEVTHQGLSSPV<br>TKSFNRGEC<br>Heavy chain (Anti-TIGIT)<br>EVQLVQSGAEVKKPGASVKVSCKASGYTFTNYWMHWVRQAPGQGLEWMGRIDPDSTGSKYNEKF<br>KTRVTMTRDTSTSTVYMELSSLRSEDTAVYYCAREGAYGYYFDYWGQGTLVTVSSASTKGPSVFPL<br>APSSKSTSGGTAALGCLVKDYFPEPVTVSWNSGALTSGVHTFPAVLQSSGLYSLSSVVTVPSSSLGTQ<br>TYICNVNHKPSNTKVDKKVEPKSCDKTHTCPPCPAPELLGGPSVFLFPPKPKDTLMISRTPEVTCVVV<br>DVSHEDPEVKFNWYVDGVEVHNAKTKPREEQYNSTYRVVSVLTVLHQDWLNGKEYKCKVSNKAL<br>PAPIEKTISKAKGQPREPQVYTLPPSRDELTKNQVSLTCLVKGFYPSDIAVEWESNGQPENNYKTTPPV<br>LDSDGSFFLYSKLTVDKSRWQQGNVFSCSVMHEALHNHYTQKSLSLSPGK<br>Light chain (Anti-TIGIT)<br>DIQMTQSPSSLSASVGDRVTITCRASENIYSYLAWYQQKPGKSPKLLIYNARTLAEGVPSRFSGSGSGT<br>DFTLTISSLQPEDFATYYCQYHSGSPLPFGGGTKVEIKRTVAAPSVFIFPPSDEQLKSGTASVVCLLNNF<br>YPREAKVQWKVDNALQSGNSQESVTEQDSKDSTYSLSSTLTLSKADYEKHKVYACEVTHQGLSSPV<br>TKSFNRGEC<br>Variable heavy chain (Anti-TIGIT)<br>EVQLVQSGAEVKKPGASVKVSCKASGYTFTNYWMHWVRQAPGQGLEWMGRIDPDSTGSKYNEKF<br>KTRVTMTRDTSTSTVYMELSSLRSEDTAVYYCAREGAYGYYFDYWGQGTLVTVSS<br>Variable light chain (Anti-TIGIT)<br>DIQMTQSPSSLSASVGDRVTITCRASENIYSYLAWYQQKPGKSPKLLIYNARTLAEGVPSRFSGSGSGT<br>DFTLTISSLQPEDFATYYCQYHSGSPLPFGGGTKVEIK

		Heavy chain (Anti-PDL1)<br>QVQLVQSGAEVKKPGASVKVSCKASGYTFTSYWMHWVRQAPGQGLEWMGRITPSSGFAMYNEKF<br>KNRVTMTRDTSTSTVYMELSSLRSEDTAVYYCARGGSSYDYFDYWGQGTTVTVSSASTKGPSVFPL<br>APSSKSTSGGTAALGCLVKDYFPEPVTVSWNSGALTSGVHTFPAVLQSSGLYSLSSVVTVPSSSLGTQ<br>TYICNVNHKPSNTKVDKKVEPKSCDKTHTCPPCPAPELLGGPSVFLFPPKPKDTLMISRTPEVTCVVV<br>DVSHEDPEVKFNWYVDGVEVHNAKTKPREEQYNSTYRVVSVLTVLHQDWLNGKEYKCKVSNKAL<br>PAPIEKTISKAKGQPREPQVYTLPPSRDELTKNQVSLTCLVKGFYPSDIAVEWESNGQPENNYKTTPPV<br>LDSDGSFFLYSKLTVDKSRWQQGNVFSCSVMHEALHNHYTQKSLSLSPGK<br>Light chain (Anti-PDL1)<br>DIVLTQSPASLAVSPGQRATITCRASESVSIHGTHLMHWYQQKPGQPPKLLIYAASKLESGVPARFSGS<br>GSGTDFTLTINPVEAEDTANYYCQQSFEDPLTFGQGTKLEIKRTVAAPSVFIFPPSDEQLKSGTASVVC<br>LLNNFYPREAKVQWKVDNALQSGNSQESVTEQDSKDSTYSLSSTLTLSKADYEKHKVYACEVTHQG<br>LSSPVTKSFNRGEC<br>Variable heavy chain (Anti-PDL1)<br>QVQLVQSGAEVKKPGASVKVSCKASGYTFTSYWMHWVRQAPGQGLEWMGRITPSSGFAMYNEKF<br>KNRVTMTRDTSTSTVYMELSSLRSEDTAVYYCARGGSSYDYFDYWGQGTTVTVSS<br>Variable light chain (Anti-PDL1)<br>DIVLTQSPASLAVSPGQRATITCRASESVSIHGTHLMHWYQQKPGQPPKLLIYAASKLESGVPARFSGS<br>GSGTDFTLTINPVEAEDTANYYCQQSFEDPLTFGQGTKLEIK
HuPL721-T2353-scFab	TIGIT×PD-L1	NA
PM-1022	TIGIT×PD-L1	NA
CX694	CD28×PD-L1	Variable heavy chain (Anti-PD-L1)<br>EVQLLESGGGEVQPGGSLRLSCAASGGIFAIKPISWYRQAPGKQREWVSTTTSSGATNYAESVKGRFT<br>ISRDNAKNTLYLQMSSLRAEDTAVYYCNVFEYWGQGTLVTVKP<br>Variable heavy chain (Anti-CD28)<br>EVQLVQSGGGLVQTGGSLRLSCAASGRMFSNYAMGWFRQAPGKEREFVAAINYRRDSADYADSVK<br>GRFTISRDNAKNTVYLEMNSLKPEDTAIYYCGFTYAGWASSRRDDYNYWGQGTQVTVKP<br>Fc domain<br>PAPGGPSVFLFPPKPKDTLMISRTPEVTCVVVDVSHEDPEVKFNWYVDGVEVHNAKTKPREEQYNST<br>YRVVSVLTVLHQDWLNGKEYKCKVSNKALPAPIEKTISKAKGQPREPQVYTLPPSRDELTKNQVSLT<br>CLVKGFYPSDIAVEWESNGQPENNYKTTPPVLDSDGSFFLYSKLTVDKSRWQQGNVFSCSVMHEAL<br>HNHYTQKSLSLSPGK<br>Linker<br>GGSGGGGS
Ab-1	CD28×PD-L1	NA
XENP-36764	CD28×PD-L1	Heavy chain-anti-PD-L1<br>EVQLLESGGGLVQPGGSLRLSCAASGFTFSSYDMSWVRQAPGKRLEWVSTISSGGSFTHYPDSVKGR<br>FTISRDNSKNTLYLQMNSLRAEDTAVYYCVRRSGDWYFDVWGAGTTVTVSSASTKGPSVFPLAPSS<br>KSTSGGTAALGCLVKDYFPEPVTVSWNSGALTSGVHTFPAVLQSSGLYSLSSVVTVPSSSLGTQTYIC<br>NVNHKPSDTKVDKKVEPKSCDKTHTCPPCPAPPVAGPSVFLFPPKPKDTLMISRTPEVTCVVVDVKH<br>EDPEVKFNWYVDGVEVHNAKTKPREEEYNSTYRVVSVLTVLHQDWLNGKEYKCKVSNKALPAPIE<br>KTISKAKGQPREPQVYTLPPSREEMTKNQVSLTCDVSGFYPSDIAVEWESDGQPENNYKTTPPVLDSD<br>GSFFLYSKLTVDKSRWEQGDVFSCSVLHEALHSHYTQKSLSLSPGK

		CD28 scFv<br>EVQLLESGGGLVQPGGSLRLSCAASGFTFSSYYMSWVRQAPGKGLEWVSTISESGDSTYYADSVKGR<br>FTISRDNSKNTLYLQMNSLRAEDTAVYYCAKSGPGLRQVGFDYWGQGTLVTVSSGKPGSGKPGSGK<br>PGSGKPGSDIQMTQSPSSLSASVGDRVTITCRASQSISSYLNWYQQKPGKAPKLLIYAASSLQSGVPSR<br>FSGSGSGTDFTLTISSLQPEDFATYYCQQSYSTPFTFGQGTKLEIKEPKSSDKTHTCPPCPAPPVAGPSV<br>FLFPPKPKDTLMISRTPEVTCVVVDVKHEDPEVKFNWYVDGVEVHNAKTKPREEQYNSTYRVVSVL<br>TVLHQDWLNGKEYKCKVSNKALPAPIEKTISKAKGQPREPQVYTLPPSREQMTKNQVKLTCLVKGF<br>YPSDIAVEWESNGQPENNYKTTPPVLDSDGSFFLYSKLTVDKSRWQQGNVFSCSVLHEALHSHYTQK<br>SLSLSPGK<br>Light chain-anti-PD-L1<br>EIVLTQSPGTLSLSPGERATLSCRVSSSISSSNLHWYQQKPGQSPRPLIYGTSNLASGVPVRFSGSGSGT<br>DYTLTISRLEPEDFAVYYCQQWSSYPLYTFGGGTKVEIKRTVAAPSVFIFPPSDEQLKSGTASVVCLLN<br>NFYPREAKVQWKVDNALQSGNSQESVTEQDSKDSTYSLSSTLTLSKADYEKHKVYACEVTHQGLSS<br>PVTKSFNRGEC
CDX-527	CD27×PD-L1	Heavy chain<br>EVQLVESGGGLVQPGGSLRLSCAASGGIISTYWMSWVRQAPGKGLEWVANIKQDGSEKYYVDSVK<br>GRFTISRDNAKNSLYLQMNSLRVEDTAMYYCARDRPVAGASALWGQGTLVTVSSASTKGPSVFPLA<br>PSSKSTSGGTAALGCLVKDYFPEPVTVSWNSGALTSGVHTFPAVLQSSGLYSLSSVVTVPSSSLGTQT<br>YICNVNHKPSNTKVDKKVEPKSCDKTHTCPPCPAPELLGGPSVFLFPPKPKDTLMISRTPEVTCVVVD<br>VSHEDPEVKFNWYVDGVEVHNAKTKPREEQYNSTYRVVSVLTVLHQDWLNGKEYKCKVSNKALP<br>APIEKTISKAKGQPREPQVYTLPPSRDELTKNQVSLTCLVKGFYPSDIAVEWESNGQPENNYKTTPPV<br>LDSDGSFFLYSKLTVDKSRWQQGNVFSCSVMHEALHNHYTQKSLSLSPGKGSSGGGGSEIVMTQSPA<br>TLSVSPGERATLSCRASQSIRSNLAWYQQKPGQAPRLLIYGASTRATGIPARFSGSGSGTEFTLTISSLQ<br>SENFAVYYCQQYNNWPLTFGCGTKVEIKGGGGSGGGGSGGGGSGGGGSQVQLVQSGAEVKKPGAS<br>VKVSCKASGYTFTGYYIHWVRQAPGQCLEWMGWINPNSGGTNSAQKFQDRVTITRVTSINTAYMEL<br>SRLRSDDTAVYFCARDRLVLPWFGEIFPDAFDIWGQGTLVTVSS<br>Light chain<br>DIQMTQSPSTLSASVGDRVTITCRASQSISGWLAWYQQKPGKAPKLLIYKASSLESGVPSRFSGSGSGT<br>EFTLTISSLQPDDFATYYCQQYYGSSRTFGQGTNVEIKRTVAAPSVFIFPPSDEQLKSGTASVVCLLNN<br>FYPREAKVQWKVDNALQSGNSQESVTEQDSKDSTYSLSSTLTLSKADYEKHKVYACEVTHQGLSSP<br>VTKSFNRGEC
28A10 x 2E4	OX40×PD-L1	Variable heavy chain (28A10)<br>QVQLQESGGGLVQAGGSLRLACTTSGGLFNIRPISWYRQPPGMQREWVATIAFGGATNYANSIKGRF<br>TASRDNAKNTVYLQMNGLKPEDTAVYYCNAFEIWGQGTQVTV<br>CDR1 variable heavy chain (28A10)<br>GGIFNIRP<br>CDR2 variable heavy chain (28A10)<br>IAFGGAT<br>CDR3 variable heavy chain (28A10)<br>NAFEI<br>Variable heavy chain (2E4)<br>QVQLQQSGGGLVQPGGSLSLSCVASGIILSHNEMRWYRQNPGKPRDLVAGITSAAYTYYGDFVKGR<br>FTISRDNAKNTAYLQMDRLNPEDTGNYYCEVSDGDNRYWGQGTQATV
IBI-327	OX40×PD-L1	anti-PD-L1/OX40 bispecific antibody peptide 1

		QVQLVQSGAEVKKPGASVKVSCKASGYTFTSYYMHWVRQAPGQGLEWMGIINPSGGSTSYAQKFQ<br>GRVTMTRDTSTSTVYMELSSLRSEDTAVYYCARDHASSSWYTTHLDLWGRGTLVTVSSASTKGPSV<br>FPLAPCSRSTSESTAALGCLVKDYFPEPVTVSWNSGALTSGVHTFPAVLQSSGLYSLSSVVTVPSSNFG<br>TQTYTCNVDHKPSNTKVDKTVERKCCVECPPCPAPPVAGPSVFLFPPKPKDTLMISRTPEVTCVVVD<br>VSHEDPEVQFNWYVDGVEVHNAKTKPREEQFNSTFRVVSVLTVVHQDWLNGKEYKCKVSNKGLPA<br>PIEKTISKTKGQPREPQVYTLPPSREEMTKNQVSLTCLVKGFYPSDIAVEWESNGQPENNYKTTPPML<br>DSDGSFFLYSKLTVDKSRWQQGNVFSCSVMHEALHNHYTQKSLSLSPGGGGGSGGGGSQVQLQESG<br>GGLVQPGGSLRLSCAASAYTISRNSMGWFRQAPGKGLEGVAAIESDGSTSYSDSVKGRFTISLDNSKN<br>TLYLEMNSLRAEDTAVYYCAAPKVGLGPRTALGHLAFMTLPALNYWGQGTLVT<br>anti-PD-L1/OX40 bispecific antibody peptide 2<br>DIQMTQSPSSLSASVGDRVTITCQASQDISNYLNWYQQKPGKAPKLLIYDASNLETGVPSRFSGSGSG<br>TDFTFTISSLQPEDIATYYCQQSANYPYTFGGGTKVEIKRTVAAPSVFIFPPSDEQLKSGTASVVCLLN<br>NFYPREAKVQWKVDNALQSGNSQESVTEQDSKDSTYSLSSTLTLSKADYEKHKVYACEVTHQGLSS<br>PVTKSFNRGEC<br>PD-L1 nanobody<br>QVQLQESGGGLVQPGGSLRLSCAASAYTISRNSMGWFRQAPGKGLEGVAAIESDGSTSYSDSVKGRF<br>TISLDNSKNTLYLEMNSLRAEDTAVYYCAAPKVGLGPRTALGHLAFMTLPALNYWGQGTLVTVSS
MEDI-1109	OX40×PD-L1	NA
BS-813	OX40×PD-L1	Heavy chain<br>QYQLYQSGAEYKKPGSSYKYSCKASGFTFSSSYISWYRQAPGQGLEWIAWIYAGTGGTSYNQKFTG<br>RATITVDESTSTAYMELSSLRSEDTAVYYCARHEGVYWYFDVWGQGTTVTVSSASTKGPSVFPLAPS<br>SKSTSGGTAALGCLVKDYFPEPVTVSWNSGALTSGVHTFPAVLQSSGLYSLSSVYTVPSSSLGTQTYI<br>CNVNHKPSNTKVDKKVEPKSCDKTHTCPPCPAPEAAGGPSVFLFPPKPKDTLMISRTPEVTCVWDVS<br>HEDPEVKFNWYVDGVEVHNAKTKPREEQYNSTYRVVSVLTVLHQDWLNGKEYKCKVSNKALPAPI<br>EKTISKAKGQPREPQVYTLPPSRDELTKNQVSLTCLVKGFYPSDIAVEWESNGQPENNYKTTPPVLDS<br>DGSFFLYSKLTVDKSRWQQGNVFSCSVMHEALHNHYTQKSLSLSPGSGGGGSGGGGSDIQMTQSPSS<br>LSASVGDRVTITCRASQDIRTYLNWYQQKPGKVPKLLIYYTSRLHSGVPSRFSGSGSGTDYTLTISSLQ<br>PEDVATYYCQQGNTLPWTFGGGTKVEIKGGGGSGGGGSGGGGSQVQLVQSGAEVKKPGSSVKVSC<br>KASGYTFTSYIMHWVRQAPGQGLEWIGYINPYNSGTKYNEKFKGRVTITSDKSTSTAYMELSSLRSE<br>DTAVYYCAHYYGSTFTMDYWGQGTTVTVSS<br>Light chain<br>EIVLTQSPATLSLSPGERATLSCSASSSVSYMHWYQQKPGQAPRPWIYDTSNLASGFPARFSGSGSGT<br>DFTLTISSLEPEDFAVYYCHQRSSYPWTFGGGTKVEIKRTVAAPSVFIFPPSDEQLKSGTASVVCLLNN<br>FYPREAKVQWKVDNALQSGNSQESVTEQDSKDSTYSLSSTLTLSKADYEKHKVYACEVTHQGLSSP<br>VTKSFNRGEC
KN-052	OX40×PD-L1	NA
EMB-09	OX40×PD-L1	NA
L52-2D7H232	OX40×PD-L1	L52-2D7H232 Heavy chain<br>DVQLVESGGGLVQPGGSLRLSCAASGFTASIYGMRWFRQAPGKGRELVAGIVDAGSATYYADSVKG<br>RFTISRDNAKNTVYLQMNSLRAEDTAVYYCARGNHEGEVGLDYWGQGTQVTVSASEFGGGGSGGG<br>GSGGGGSDVQLVESGGGLVQPGGSLRLSCAASGFTASIYGMRWFRQAPGKGRELVAGIVDAGSATY<br>YADSVKGRFTISRDNAKNTVYLQMNSLRAEDTAVYYCARGNHEGEVGLDYWGQGTQVTVSASGG<br>GGSGGGGSGGGGSLEQVQLVESGGGLVQPGGSLRLSCTVSGIDLSSYDMTWVRQAPGKGLEYIGYIS<br>YVSRTYYADSVKGRFTISKDTSKNTVYLQMNSLRAEDTAVYYCARDRPDGAATNLWGQGTLVTVS

		SASTKGPSVFPLAPSSKSTSGGTAALGCLVKDYFPEPVTVSWNSGALTSGVHTFPAVLQSSGLYSLSS<br>VVTVPSSSLGTQTYICNVNHKPSNTKVDKKVEPKSCDKTHTCPPCPAPELLGGPSVFLFPPKPKDTLMI<br>SRTPEVTCVVVDVSHEDPEVKFNWYVDGVEVHNAKTKPREEQYNSTYRVVSVLTVLHQDWLNGKE<br>YKCKVSNKALPAPIEKTISKAKGQPREPQVYTLPPSRDELTKNQVSLTCLVKGFYPSDIAVEWESNGQ<br>PENNYKTTPPVLDSDGSFFLYSKLTVDKSRWQQGNVFSCSVMHEALHNHYTQKSLSLSP<br>L52-2D7H232 Light chain<br>DIQMTQSPSTLSASVGDRVTITCQSSQNVYSNNRLSWYQQKPGKAPKLLIYWTSFLASGVPSRFSGSG<br>SGTEFTLTISSLQPDDFATYYCAGGYSGNLYTFGQGTKLEIKRTVAAPSVFIFPPSDEQLKSGTASVVC<br>LLNNFYPREAKVQWKVDNALQSGNSQESVTEQDSKDSTYSLSSTLTLSKADYEKHKVYACEVTHQG<br>LSSPVTKSFNRGEC<br>L52-2D7H232 Variable light chain<br>DIQMTQSPSTLSASVGDRVTITCQSSQNVYSNNRLSWYQQKPGKAPKLLIYWTSFLASGVPSRFSGSG<br>SGTEFTLTISSLQPDDFATYYCAGGYSGNLYTFGQGTKLEIKR<br>CDR1 variable heavy chain region (Anti-OX40)<br>GFTASIYGMR<br>CDR2 variable heavy chain region (Anti-OX40)<br>GIVDAGSATYYAD<br>CDR3 variable heavy chain region (Anti-OX40)<br>GNHEGEVGLDY
ES-101	CD137×PD-L1	NA
MCLA-145	CD137×PD-L1	NA
ATG-101	CD137×PD-L1	Heavy chain<br>QVQLVQSGAEVKKPGSSVKVSCKASRGPFSTYAISWVRQAPGQGLEWMGRIIPILGIANYAQKFQGR<br>VTITADKSTSTAYMELSSLRSEDTAVYYCARTMEGYGFGNFDYWGQGTLVTVSSASTKGPSVFPLAP<br>SSKSTSGGTAALGCLVKDYFPEPVTVSWNSGALTSGVHTFPAVLQSSGLYSLSSVVTVPSSSLGTQTY<br>ICNVNHKPSNTKVDKKVEPKSCDKTHTCPPCPAPELLGGPSVFLFPPKPKDTLMISRTPEVTCVVVDV<br>SHEDPEVKFNWYVDGVEVHNAKTKPREEQYASTYRVVSVLTVLHQDWLNGKEYKCKVSNKALPAP<br>IEKTISKAKGQPREPQVYTLPPSREEMTKNQVSLTCLVKGFYPSDIAVEWESNGQPENNYKTTPPVLD<br>SDGSFFLYSKLTVDKSRWQQGNVFSCSVMHEALHNHYTQKSLSLSPGGGGSGGGGSGGGGSQVQLV<br>ESGGGLVQPGGSLRLSCAASGFTFSSYAMSWVRQAPGKGLEWVSAISGSGGSTYYADSVKGRFTISR<br>DNSKNTLYLQMNSLRAEDTAVYYCAKTNWGPSDAFDIWGQGTMVTVSSASTGGGGSGGGGSGGG<br>GSQSVLTQPPSASGTPGQRVTISCSGSTSDIGSYSVNWYQQLPGTAPKLLIYSNNQRPSGVPDRFSGSK<br>SGTSASLAISGLQSEDEADYYCAAWDDSLNGYVFGTGTKLTVLG<br>Light chain<br>QSALTQPASVSGSPGQSITISCTGTVSEVGGYNEVSWYQQHPGKAPKLMIYGNSNRPSGVSNRFSGSK<br>SGNTASLTISGLQAEDEADYYCQSYDSSLSGTVFGGGTKLTVLGQPKAAPSVTLFPPSSEELQANKAT<br>LVCLISDFYPGAVTVAWKADSSPVKAGVETTTPSKQSNNKYAASSYLSLTPEQWKSHRSYSCQVTHE<br>GSTVEKTVAPTECS
ABL-503	CD137×PD-L1	NA
FS-222	CD137×PD-L1	NA
S-095012	CD137×PD-L1	NA
AP-203	CD137×PD-L1	NA
4-1BB<br>(20H4.9) x	CD137×PD-L1	Heavy chain<br>DIQMTQSPSSLSASVGDRVTITCRASQDVSTAVAWYOOKPGKAPKIAIYSASFLYSGVPSRFSGSGSG

PD-L1		TQFTLTISSLQPEDFATYYCOQYLYHPATFGOGTKVEIKSSASTKGPSVFPLAPSSKSTSGGTAALGCL<br>VKDYFPEPVTVSWNSGALTSGVHTFPAVLQSSGLYSLSSVVTVPSSSLGTQTYICNVNHKPSNTKVDK<br>KVEPKSCDGGGGSGGGGSQVQLQQWGAGLLKPSETLSLTCAVYGGSFSGYYWSWIRQSPEKGLEWI<br>GEINHGGYVTYNPSLESRVTISVDTSKNQFSLKLSSVTAADTAVYYDARDYGPGNYDWYFDLWGRG<br>TLVTVSSASTKGPSVFPLAPSSKSTSGGTAALGCLVEDYFPEPVTVSWNSGALTSGVHTFPAVLQSSG<br>LYSLSSVVTVPSSSLGTQTYICNVNHKPSNTKVDEKVEPKSCDKTHTCPPCPAPEAAGGPSVFLFPPKP<br>KDTLMISRTPEVTCVVVDVSHEDPEVKFNWYVDGVEVHNAKTKPREEQYNSTYRVVSVLTVLHQD<br>WLNGKEYKCKVSNKALGAPIEKTISKAKGQPREPQVYTLPPCRDELTKNQVSLWCLVKGFYPSDIAV<br>EWESNGQPENNYKTTPPVLDSCGSFFLYSKLTVDKSRWQOGNVFSCSVMHEALHNHYTOKSLSLSP<br>Light chain<br>EVQLVESGGGLVQPGGSLRLSCAASGFTFSDSWIHWVRQAPGKGLEWVAWISPYGGSTYYADSVKG<br>RFTISADTSKNTAYLQMNSLFAEDTAVYYCARRHWPGGFDYWGQGTLVTVSSASVAAPSVFIFPPSD<br>ECILKSGTASVVCLLNNFYPREAKVOWKVDNALOSGNSQESVTEODSKDSTYSISSTLTLSKADYEK<br>HKVYACEVTHOGLSSPVTKSFNRGEC
GEN-1046	CD137×PD-L1	NA
LBL-024	CD137×PD-L1	Variable heavy chain (Anti-4-1BB)<br>QVQLQESGGGLVQPGGSLRLSCAVSGFTFSSYAMHWVRQAPGKCLEWVAVISYDGSKKWYADSVK<br>GRFTISRDNSKNTLYLQMNSLRAEDTAVYYCARNQGSGSYLYYYYMDVWGKGTTVTVSS<br>CDR1 variable heavy chain (Anti-4-1BB)<br>SYAMH<br>CDR2 variable heavy chain (Anti-4-1BB)<br>VISYDGSKKWYADSVKG<br>CDR3 variable heavy chain (Anti-4-1BB)<br>NQGSGSYLYYYYMDV<br>Variable light chain (Anti-4-1BB)<br>QSALTQPRSVSGSPGQSVTISCTGTSSDVGGYNYVSWYQQLPGKAPKVIIYEVSNRPSGVSNRFSGSK<br>SGNTASLTISGVQSEDEADYYCSSYTSSSTFYVFGCGTQLTVL<br>CDR1 variable light chain (Anti-4-1BB)<br>TGTSSDVGGYNYVS<br>CDR2 variable light chain (Anti-4-1BB)<br>EVSNRPS<br>CDR3 variable light chain (Anti-4-1BB)<br>SSYTSSSTFYV<br>Variable heavy chain (Anti-PD-L1)<br>EVQLQESGPGLVKPSQTLSLTCTVSGDSFSSGYWNWIRQHPGKGLEYIGYVSYTGSTYYIPSLKSRVTI<br>SRDTSKNQFSLKLSSVTAADTAVYYCAGYRDWLHGYFDYWGQGTTVTVSS<br>CDR1 variable heavy chain (Anti-PD-L1)<br>SGYWN<br>CDR2 variable heavy chain (Anti-PD-L1)<br>YVSYTGSTYYIPSLKS<br>CDR3 variable heavy chain (Anti-PD-L1)<br>YRDWLHGYFDY<br>Variable light chain (Anti-PD-L1)<br>DIQMTQSPSSLSASVGDRVTITCKASQNVMDNVAWYQQKPGKAPKRLIYSASYRFSGVPSRFSGSGS

		GTEFTLTISSLQPEDFATYYCQQYNGYPLTFGQGTKLEIK<br>CDR1 variable light chain (Anti-PD-L1)<br>KASQNVMDNVA<br>CDR2 variable light chain (Anti-PD-L1)<br>SASYRFS<br>CDR3 variable light chain (Anti-PD-L1)<br>QQYNGYPLT
9EN-FM	CD137×PD-L1	First polypeptide chain<br>QVQLVQSGAEVKKPGASVKVSCKASGYTFTSYWMHWVRQAPGQGLEWMGRIGPNSGFTSYNEKF<br>KNRVTMTRDTSTSTVYMELSSLRSEDTAVYYCARGGSSYDYFDYWGQGTTVTVSSGGGGSGGGGS<br>GGGGSDIVLTQSPASLAVSPGQRATITCRASESVSIHGTHLMHWYQQKPGQPPKLLIYAASNLESGVP<br>ARFSGSGSGTDFTLTINPVEAEDTANYYCQQSFEDPLTFGQGTKLEIKGGGGSGGGGSGGGGSGGGG<br>SEVQLVQSGAEVKKPGSSVKVSCKASGYTFTSYGLNWVRQAPGQRLEWMGYINPGSGYTKYNEKF<br>EGRVTITADKSTSTAYMELSSLRSEDTAVYFCARWGLGRNWNFAVWGQGTLVTVSSASTKGPSVFP<br>LAPSSKSTSGGTAALGCLVKDYFPEPVTVSWNSGALTSGVHTFPAVLQSSGLYSLSSVVTVPSSSLGT<br>QTYICNVNHKPSNTKVDKKVEPKSCDKTHTCPPCPAPEAAGGPSVFLFPPKPKDTLMISRTPEVTCVV<br>VDVSHEDPEVKFNWYVDGVEVHNAKTKPREEQYNSTYRVVSVLTVLHQDWLNGKEYKCKVSNKA<br>LGAPIEKTISKAKGQPREPQVYTLPPSREEMTKNQVSLTCLVKGFYPSDIAVEWESNGQPENNYKTTP<br>PVLDSDGSFFLYSKLTVDKSRWQQGNVFSCSVMHEALHNHYTQKSLSLSPGK<br>Second polypeptide chain<br>DIVMTQSPDSLAVSLGERATINCRASESVDSYGNTFMHWYQQKPGQPPKLLIYRASQLESGVPDRFS<br>GSGSGTDFTLTISSLQAEDVAVYYCQQSNEDPLTFGQGTKLEIKRTVAAPSVFIFPPSDEQLKSGTASV<br>VCLLNNFYPREAKVQWKVDNALQSGNSQESVTEQDSKDSTYSLSSTLTLSKADYEKHKVYACEVTH<br>QGLSSPVTKSFNRGEC
PM-1003	CD137×PD-L1	NA
QL-301	CD137×PD-L1	NA
MF6797xMF77<br>02	CD137×PD-L1	Variable heavy chain (Anti-CD137)<br>QITLKESGPTLVKPTQTLTLTCTFSGFSLSTTGVGVNWIRQPPGEALEWLALIYWNDDTYYSPSLKSRL<br>TITKDTSKNQVVLTMTNMDPVDTATYYCAHEGIIGFLGGNWFDPWGQGTLVTVSS<br>CDR1 variable heavy chain (Anti-CD137)<br>TTGVGVN<br>CDR2 variable heavy chain (Anti-CD137)<br>LIYWNDDTYYSPSLKS<br>CDR3 variable heavy chain (Anti-CD137)<br>EGIIGFLGGNWFDP<br>CDR1 variable heavy chain (Anti-PD-L1)<br>DRKYVTNWVFAEDFQH<br>CDR2 variable heavy chain (Anti-PD-L1)<br>WINPNTGNPTYAQGFTG<br>CDR3 variable heavy chain (Anti-PD-L1)<br>NYAIN<br>Variable heavy chain (Anti-PD-L1)<br>QVQLVQSGSELKKPGASVKVSCKASGYTFTNYAINWVRQAPGQGLEWMGWINPNTGNPTYAQGFT<br>GRFVFSLDTSVSTAYLQISSLKAEDTAVYYCARDRKYVTNWVFAEDFQHWGRGTLVTVSS

BH-3120	CD137×PD-L1	NA
euPD-1×94 kvt LHC 218	CD137×PD-L1	FLESPDRPWNAPTFSPALLLVAEGDNATFTCSFSNASESFHVVWHRESPSGQTDTLAAFPEDRSQPGQ<br>DHRFRVTRLPNGRDFHMSVVRAQRNDSGTYVCGVISLAPKIQIKESLRAELRVTERRAEVPTAHPSPS<br>PRPAGQFQTLVVGASGGGGSGGGGSDKTHTCPPCPAPEAAGGPSVFLFPPKPKDTLMISRTPEVTCVV<br>VDVSHEDPEVKFNWYVDGVEVHNAKTKPREEQYNSTYRVVSVLTVLHQDWLNGKEYKCAVSNKA<br>LPAPIEKTISKAKGQPREPQVYTLPPSREEMTKNQVSLTCLVKGFYPSDIAVEWESNGQPENNYKTTP<br>PVLDSDGSFFLYSKLTVDKSRWQQGNVFSCSVMHEALHNHYTQKSLSLSPGGGGGSGGGGSDIVMT<br>QSPAFLSVTPGEKVTITCRASQTISDYLHWYQQKPDQAPKLLIKYASQSISGIPSRFSGSGSGTDFTFTIS<br>SLEAEDAATYYCQDGHSWPPTFGQGTKLEIKGSTSGSGKPGSGEGSTKGQVQLVQSGAEVKKPGAS<br>VKLSCKASGYTFSSYWMHWVRQAPGQGLEWIGEINPGNGHTNYNEKFKSRVTMTRDTSTSTAYME<br>LSSLRSEDTAVYYCARSFKTARAFAYWGQGTLVTVSS
PR-004270	CD137×PD-L1	Heavy chain<br>DIQMTQSPSTLSASVGDRVTVTCRASQSIYIWLAWYQQKPGKAPNLLIYKASSLETGVPSRFSGSGSG<br>TEFTLTISSLQPDDFATYYCQQYYGSSRTFGQGTKVEIKRTVAAPSVFIFPPSDEQLKSGTASVVCLLN<br>NFYPREAKVQWKVDNALQSGNSQESVTEQDSKDSTYSLSSTLTLSKADYEKHKVYACEVTHQGLSS<br>PVTKSFNRGECEPKSSDKTHTPPPPPEVQLVESGGGVVQPGGSLRLSCAASGFTFSNYAMTWVRQAP<br>EKGLEWVSSISGSGVSTYYADSVKGRFTISRDNSKNTLYLQMTRLTAEDTAVYFCAKEGSSETDDHY<br>YNVDVWGQGTTVTVSSEPKSSDKTHTCPPCPAPEAAGGPSVFLFPPKPKDTLMISRTPEVTCVVVDVS<br>HEDPEVKFNWYVDGVEVHNAKTKPREEQYNSTYRVVSVLTVLHQDWLNGKEYKCKVSNKALPAPI<br>EKTISKAKGQPREPQVYTLPPSREEMTKNQVSLTCLVKGFYPSDIAVEWESNGQPENNYKTTPPVLDS<br>DGSFFLYSKLTVDKSRWQQGNVFSCSVMHEALHNHYTQKSLSLSPGK<br>Light chain<br>EVQLVESGGGLVQPGGSLRLSCAASGFTFSSYWMSWVRQAPGKGLEWVANIKQEGSEKYYVDSVK<br>GRFTISRDNAKNSLYLQMNSLRAEDTAVYYCARDRAVAGAFDIWGQGTMVTVSSASTKGPSVFPLA<br>PSSKSTSGGTAALGCLVKDYFPEPVTVSWNSGALTSGVHTFPAVLQSSGLYSLSSVVTVPSSSLGTQT<br>YICNVNHKPSNTKVDKKVEPKSC
HK-010	CD137×PD-L1	NA
1923Ab18	CD137×PD-L1	Light chain<br>EIVMAQSPATLSLSPGERATLSCRASQSVSSSYLSWFQQKPGQAPRLLIYGASTRATGIPARFGGSGSG<br>TDFTLTISSLQPEDFAVYYCQQDYNLPYTFGQGTKLEIKRTVAAPSVFIFPPSDEQLKSGTASVVCLLN<br>NFYPREAKVQWKVDNALQSGNSQESVTEQDSKDSTYSLSSTLTLSKADYEKHKVYACEVTHQGLSS<br>PVTKSFNRGEC<br>Heavy chain<br>QVQLVQSGAEVKKPGASVKVSCKASGYTFTSYDINWVRQATGQGLEWMGWMNPNSGITGYAQRF<br>QGRVTMTRDTSISTAYMELSSLRSEDTAVYYCARHRWGKGYLDLWGRGTLVTVSSASTKGPSVFPL<br>APSSKSTSGGTAALGCLVKDYFPEPVTVSWNSGALTSGVHTFPAVLQSSGLYSLSSVVTVPSSSLGTQ<br>TYICNVNHKPSNTKVDKKVEPKSCDKTHTCPPCPAPEAAGGPSVFLFPPKPKDTLMISRTPEVTCVVV<br>DVSHEDPEVKFNWYVDGVEVHNAKTKPREEQYNSTYRVVSVLTVLHQDWLNGKEYKCKVSNKAL<br>PAPIEKTISKAKGQPREPQVYTLPPSRDELTKNQVSLTCLVKGFYPSDIAVEWESNGQPENNYKTTPPV<br>LDSDGSFFLYSKLTVDKSRWQQGNVFSCSVMHEALHNHYTQKSLSLSPGGGGSGGGGSGGGGSQVQ<br>LVESGGGVVQPGRSLRLSCAASGFTFSNYGMHWVRQAPGKCLEWVAIIWYDGINKYYADSVKGRFT<br>ISRDNSKNTLYLQMNSLKAEDTAVYYCARDQDYDILTGYYKEDYFDFWGQGTLVTVSSGGGGSGG<br>GGSGGGGSGGGGSSYELTQPPSVSVSPGQTARITCSGDALPKKYAYWYQQKSGQAPVLVIYEDSKRP<br>SGIPERFSGSSSGTMATLTISGAQVEDEADYYCYSTDSSGNRVFGCGTKLTVL

KY-1055	ICOS×PD-L1	Variable heavy chain (Anti-ICOS)<br>EVQLVESGGGVVRPGGSLRLSCVASGVTFDDYGMSWVRQAPGKGLEWVSGINWNGGDTDYSDSV<br>KGRFTISRDNAKNSLYLQMNSLRAEDTALYYCARDFYGSGSYYHVPFDYWGQGILVTVSS<br>Variable light chain (Anti-ICOS)<br>EIVLTQSPGTLSLSPGERATLSCRASQSVSRSYLAWYQQKRGQAPRLLIYGASSRATGIPDRFSGDGSG<br>TDFTLSISRLEPEDFAVYYCHQYDMSPFTFGPGTKVDIK<br>CDR1 variable heavy chain (Anti-ICOS)<br>GVTFDDYG<br>CDR2 variable heavy chain (Anti-ICOS)<br>INWNGGDT<br>CDR3 variable heavy chain (Anti-ICOS)<br>ARDFYGSGSYYHVPFDY<br>CDR1 variable light chain (Anti-ICOS)<br>QSVSRSY<br>CDR2 variable light chain (Anti-ICOS)<br>GAS<br>CDR3 variable light chain (Anti-ICOS)<br>HQYDMSPFT
